# Body proportions for the facilitation of walking, running and flying: the case of partridges

**DOI:** 10.1186/s12862-018-1295-x

**Published:** 2018-11-26

**Authors:** Jesús Nadal, Carolina Ponz, Antoni Margalida

**Affiliations:** 1 Department of Animal Science, Division of Wildlife, Faculty of Life Sciences and Engineering, University of Lleida, Lleida, Spain; 2 Institute for Game and Wildlife Research, IREC (CSIC-UCLM-JCCM), 13005 Ciudad Real, Spain; 3 Division of Conservation Biology, Institute of Ecology and Evolution, University of Bern, Bern, Switzerland

**Keywords:** Body proportions, Body relations, Partridge mobility, Run-fly, Size balance, Walk-run

## Abstract

**Background:**

Predation is one of the most important natural selection forces. Prey species can optimize feeding behavior and escape from predators based on mobility conditioned by body proportions. With age, mobility capacity increases and individuals are more efficient in finding resources and safety (e.g., food and refuge). Birds’ mobility is driven by the dimensions, of the head and torso, as well as the extremities and flight feathers. To assess the relationship between body traits and to understand how body proportions are organized in wild Red-legged partridges (*Alectoris rufa*), we used biometric data from nearly 14,000 individuals, obtained during a long-term study (1988–2011) on a wild population.

**Results:**

We used GLMs and regressions to model the relationship between body mass and the size of body parts. We found that wing length was the morphological part best explained by other body trait measures. Wing length models were better predictors in juveniles than in adults and in females than in males. Wing length and feather length, mass and total length are the most strongly related parts; mass and wing length, total length and feather length are moderately related. The association between mass and wing length is intermediated by feather length and total length.

**Conclusions:**

Social inclusion, feeding and predator evasion may be affected by body structure intermediated by mobility and health. Our results suggest that proportions of the body, extremities and flight feathers drive mobility which is intimately associated with ecology, biological efficiency, health and physical optimization. Our findings showed that wing size was strongly allied to other body part measurements, enhancing the importance of body structure conformation for flight. Our study highlights the scaled relationship of body structure among age-sex classes and its relevance to social cohesion, flock movement and the balance between predation and starvation.

**Electronic supplementary material:**

The online version of this article (10.1186/s12862-018-1295-x) contains supplementary material, which is available to authorized users.

## Background

Body structure is related to motion and function, as well as to diseases and dysfunctions [1, 2]. Body proportions are linked to the interaction of many developmental, ecological, and biomechanical factors [3–5], and can serve as an indicator of an animal’s health. For example, in humans and dogs, obesity may indicate disease and the loss of mobility [6–8]. Body structure is defined as the regular proportions of different parts of an animal. These proportions are frequently studied through the power relations between body parts (i.e., scaling, isometry and allometry) [9]. In this sense, a simplified model for partially assessing body structure is body condition, an index used frequently to evaluate animal quality [10]. On the other hand, body structure is a much broader concept because it is directly connected to form and function, which can be considered from different perspectives (e.g. functional anatomy, functional morphology, biomechanics [11, 12]).

The relationship between form and function in animals is supported by proportions of different morphological parts, which change according to species, sex, and age classes [13]. For example, locomotor performance can be influenced by age, sex, breed and species [14, 15]. Form and function are allied with species ecology, and thus body traits can explain how the animals obtain food or escape from predators [16, 17]. In this regard, prey species that live in groups must develop strategies to feed and ward off predators according to their mobility [18, 19].

The morphology of the wings, legs and tail is influenced by the constraints of locomotion and feeding behavior [20–23]. For example, some birds eat insects while flying, others eat by perching on trees or bushes, and others eat seeds while walking. Moreover, flight and feeding strategies change between subspecies. Similarly, the body is multiply scaled among: (i) individuals of distinct age-sex classes to facilitate social hierarchy, cohesion and coordination [24], (ii) body parts to improve mechanical and physiological functions [25], and (iii) mass and length of primary feathers for better flight [26].

Red-legged partridges (*Alectoris rufa* Linnaeus, 1758) are ground-dwelling, precocial birds and specialists in walking and running. The partridges of the genus *Alectoris* are an ideal model for the study of locomotion including: walking, running wing-assisted running and the origin of the flight [20, 27, 28], although different locomotion types can have opposite body requirements [11]. Here, we assessed models of wild partridge traits, to understand the potential influence of body structure on partridge walking, running and flying. We took advantage of biometric data obtained during a long-term study of a wild partridge population inhabiting southern Spain. Our objectives were to: identify relationships between body parts through models of traits, and interpret how body structure relates to mobility for finding food and evading predators.

We argue that body structure can affect survival, based on a causal chain: morphology is related to mobility, mobility is related to escape, escape is related to predation [29]. We predict that the wing length model is more related to flight, the total length model is more related to walking-running, and the mass model is related to both locomotion types. Juvenile females should show a better fit for the wing length model than other groups because they have a better surface/mass index.

## Methods

### Study area

We examined wild partridges collected during hunts from “Las Ensanchas”, a small game hunting estate in the Jabalón River basin in Ciudad Real, Spain (38°39’ N, 3°13’ W, 790–840 m a.s.l.). The area contains a mosaic of cereal crops, fallow, natural pastures and scrubland with scattered holm oaks (*Quercus ilex*). Overall, 75% of the estate is covered by herbaceous vegetation and 25% by shrub land. The landscape is Mediterranean dehesa (open woods with pastures and cultivated land, see details in [30]).

### Partridge sex, age and mobility

Red-legged partridges exhibit sexual size dimorphism, with males being larger than females by a factor of 1.2% body mass [31]. These differences in size between sexes imply differences in the development of body structure. After hatching, chick mass is around 3.3% of their final adult mass, increasing rapidly during phases of chick (at 84 days old, 71%) and juvenile (at 122 days old, 95.9%) growth, but much less so during maturation (at 488 days old, 100%) [32].

The physical and behavioral development of chicks is shaped by feeding and evading predation, involving patterns and mechanisms of mobility to improve survival [33, 34]. In this sense, chicks develop rapid walking and running abilities with several developmental improvements: stride frequency [25], erect limbs and slender skeletal features, combined use of legs and wings [27], decreased stance duration (the duration that a given foot is in contact with the ground) and increased swing duration (the duration that a given foot is not in contact with the ground) [35]. Furthermore, social interactions allow chicks to acquire maneuverability and unpredictability in their trajectories, and guard protection while foraging and resting [17].

### Hypothesis on body proportions and ecology

**Fig. 1:**
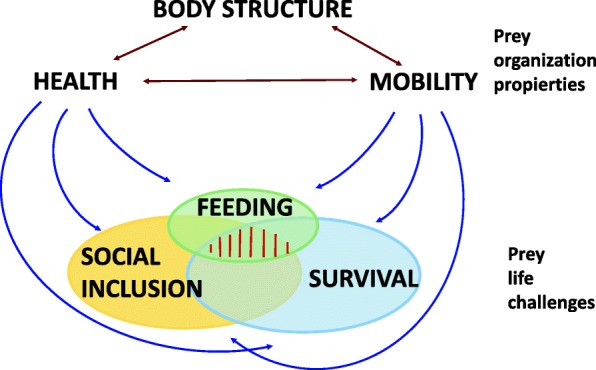
Body structure as a driver of partridge survival. The body part proportions influences health and mobility, both affects the relations among conspecifics, heterospecifics and feeding behavior

The parts of the body are structured to produce mobility [36]. Partridges forage by walking and escape from potential predators by running, jumping and then flying [30]. Walking (< 0.75 ms^-1^), running (< 1.67 ms^-1^) and flying (> 1.67 ms^-1^) appear gradually in chick development, and with age they gain speed [37]. The trunk inclination decreases from walking to being parallel to the ground in flying (Additional file 1). Body structure can affect both the health and mobility of individuals, influencing social acceptance, feeding and survival (Fig. 1). In previous studies we found scaling between mass and body length, wing length [38], 8th, 9th 10th feather length, and surface index (body length x wing length) [39], with bivariate approximations of multivariate phenomena. Scaling reflects physical constraints and natural selection based on a conflict between terrestrial and aerial locomotion due to multiobjective optimization [11] of body structure. We predict that *Alectoris* body structure according to age and sex classes are consistent with social inclusion and ecology (starvation-predation) to gain biological efficiency (future viability). Accordingly, body proportions should respond to the behavioral ecology of the species. We interpret models of body parts in terms of walking, running and flight for feeding and predator evasion.

### Data collection

Between 1998 and 2011, we studied wild partridges collected during hunts from an increasing population of birds in central Spain. Age was determined by an examination of primary feathers and sex by spur characteristics [30]. Individuals recovered in the field were weighed using a digital balance with a minimum increment of 1 g and body length was measured from beak to tail. A wing was taken from all birds (cut through the ulna-radius) and prepared for study in the laboratory.

**Fig. 2:**
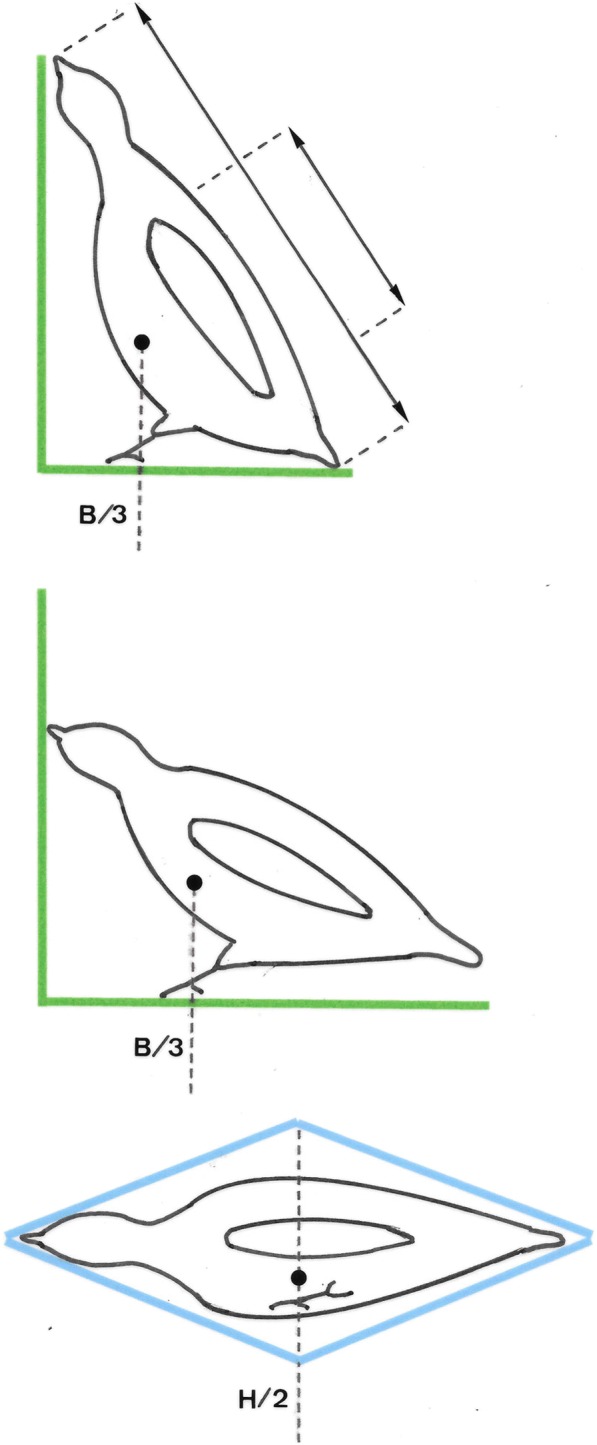
Estimation of simplified CoM (Center of Mass) for walking and flying according to geometric features of Red-legged partridge body section

Wings were dried for 15 days at 40 °C. We recorded wing length (length of wrist to wingtip with the wing folded), and length of the 8th, 9th and 10th primary feathers (length of tip to integument insertion), all to the nearest 0.5 mm. We estimated the center of mass (CoM) position for walking and flying partridges. We assumed that the body plan of a walking partridge can be simplified in the lateral view as a right triangle. Thus, the total length is the hypotenuse (h), the body projection to the floor is the base (b), and the body projection to the wall is the height (a). For a walking partridge the calculation was b = body length-wing length, and for a running partridge b = body length – (wing length × 0.9). We calculated the CoM as b/3. Partridges in flight can be simplified in the lateral view as a diamond and their vertical axis (h/2) indicates the CoM (Fig. 2).

### Statistical analyses

We used Generalized Linear Models (GLMs) to assess the organization of body parts (Y = mass, total length or wing length) in (a) overall partridge data, (b) age or sex groups, and (c) age-sex classes. We analyzed partridges generally (all partridge) and in particular (one class of age and sex, e.g. juvenile female). We used GLMs with a normal distribution and identity as a link function to explain mass, total length and wing length [40]. The number of variables with significant effects were used to assess different models, as well as the log of utility of each variable for the model. We checked whether one explained variable produced a better fit for one age group than another, or for one sex vs the other, or for one age-sex class vs the other. [41]. We performed multiple and simple regression models with body variables in the overall partridge data. We used covariance analyses of body parts to search for equality or inequality (significant interaction) of slopes among age-sex classes. We calculated variance inflation factor (VIF) to test for multicollinearity, and to verify the residual distribution and autocorrelation (Additional files 2, 3, 4, 5, 6, 7 and 8). We used JMP12 to statistically analyze the studied variables [42].

## Results

**Fig. 3:**
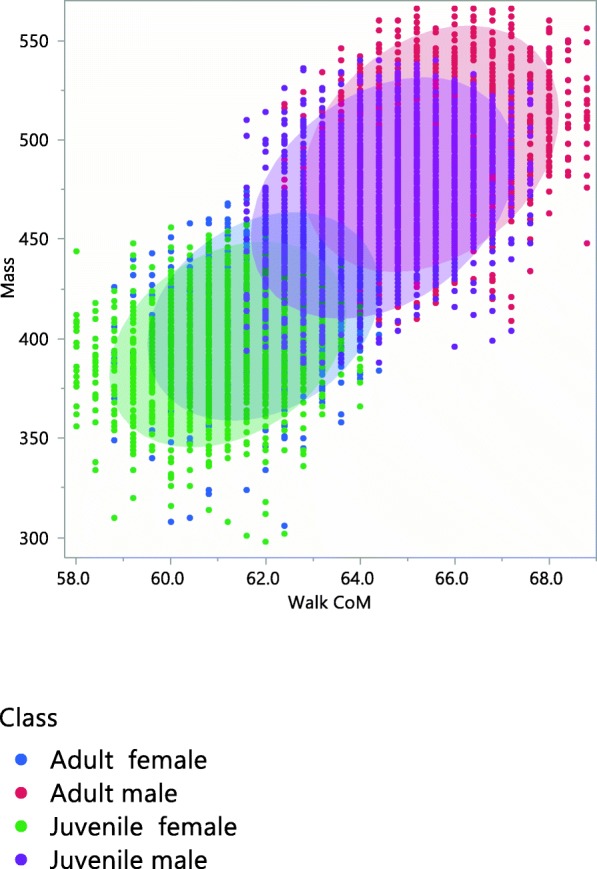
Scaled relationship between mass (g) and simplified CoM (Center of Mass) for walking with respect to the reference point in the Red-legged partridge according to age and sex class. 90% confidence ellipses for age-sex classes

**Fig. 4:**
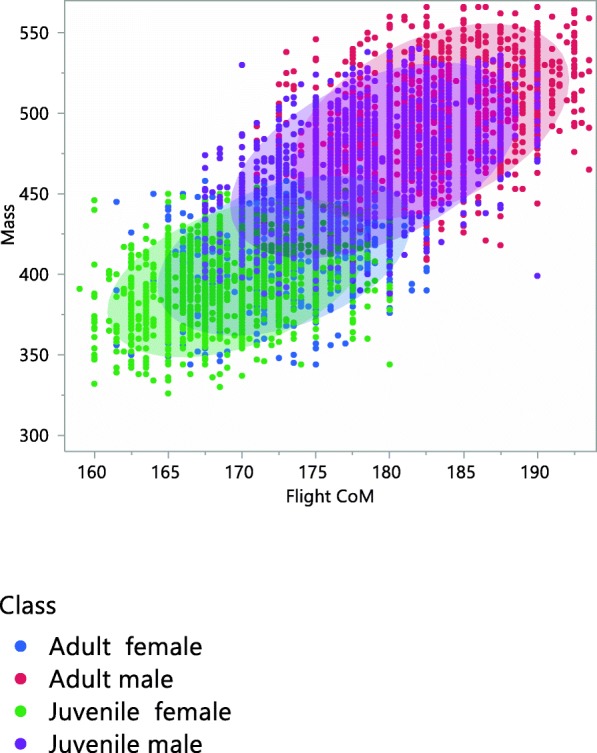
Scaled relationship between mass (g) and simplified CoM (Center of Mass) for flight with respect to the reference point in Red-legged partridge according to age and sex class. 90% confidence ellipses for age-sex classes

Over 14 years, we examined 13,814 wild partridges, 77% of which were captured in October, 20% in November and 3% in December throughout the course of this study. Of the partridges that were examined: 9938 (72%) were used for mass measurements, 7529 (54%) for total length, 11,539 (83.5%) for wing length, 11,844 (85.7%) for 10th primary length, 13,011 (94.2%) for 9th primary length and 10,696 (61.4%) for 8th primary length. The relative CoM position is different for walking and flying. The CoM changes with the scaling of size according to age and sex (Table1, Figs. 2, 3 and 4 and Additional file 9) as the larger the size, the greater the distance of CoM from a reference point (position).

**Table 1:** Estimation of CoM (Center of Mass) position from reference point (cm) in walking and flying partridges according to age and sex class

	Juvenile female	Adult female	Juvenile male	Adult male
CoM in walking	6.12	6.19	6.44	6.54
CoM in flying	16.98	17.28	17.90	18.28

The wing length model revealed significant effects for all parameters considered (mass, total length, 8th, 9th and 10th primary length). The total length model was not significant for the 10th primary length effect, and the mass model was not significant for the 9th primary length effect (Table 2). Similar results were obtained in age (old and young), and in sex (female and male) models (Table 3), and in age-sex class models (Table 4, Additional files 2 and 3). The effects of different parameters were distinct in each model (log utility). Mass and total length were strongly related, while mass and wing length were moderately related. Similarly wing length and feather length were strongly related, while total length and feather length were moderately related. The association of mass and wing length was intermediated by feather length and total length.

**Table 2:** Effects and log utility of models. Mass, total length and wing length explained by effects of wing length, total length, mass, length of 8th, 9th, 10th primaries

	Mass		Total length		Wing length	
	Effects	Log Utility	Effects	Log Utility	Effects	Log Utility
Mass	–	–	**0.0001**	231.2	**0.0001**	88.5
Total length	**0.0001**	231.2	–	–	**0.0013**	2.9
Wing length	**0.0001**	88.5	**0.0013**	2.9	–	–
10 length	**0.0001**	10.9	0.56	0.2	**0.0001**	18.7
9 length	0.96	0.0	**0.0001**	10.6	**0.0001**	8.0
8 length	**0.004**	2.4	**0.0005**	3.3	**0.0001**	179.2

Significant results appear in bold type

**Table 3:** Effects and log utility of models. Age or sex of mass for total length and wing length explained by effects of wing length, total length, mass, length of 8th, 9th, 10th primaries

	Mass		Total length		Wing length	
	Effects	Log Utility	Effects	Log Utility	Effects	Log Utility
Adult						
Mass	–	–	**0.0001**	134.4	**0.0001**	45.6
Total length	**0.0001**	134.4	–	–	**0.02**	1.8
Wing length	**0.0001**	45.6	**0.016**	1.8	–	–
10 length	**0.0001**	5.0	0.34	0.5	**0.02**	1.7
9 length	0.31	0.5	0.20	0.7	0.2	0.7
8 length	**0.03**	1.5	**0.004**	2.4	**0.001**	60.4
Juvenile						
Mass	–	–	**0.0001**	100.6	**0.0001**	37.9
Total length	**0.0001**	100.6	–	–	**0.01**	2.0
Wing length	**0.0001**	37.9	**0.01**	2.0	–	–
10 length	0.01	2.0	0.92	0.04	**0.004**	2.4
9 length	0.13	0.9	**0.007**	2.1	**0.003**	2.5
8 length	**0.02**	1.7	**0.005**	2.8	**0.0001**	80.0
Female						
Mass	–	–	**0.0001**	48.6	**0.0001**	11.0
Total length	**0.0001**	48.7	–	–	**0.04**	1.4
Wing length	**0.0001**	11.0	**0.04**	1.4	–	–
10 length	0.25	0.6	**0.03**	1.5	**0.02**	1.7
9 length	0.23	0.6	**0.0001**	9.0	**0.04**	1.4
8 length	**0.01**	2.0	0.38	0.4	**0.001**	39.4
Male						
Mass	–	–	**0.0001**	95.6	**0.0001**	26.0
Total length	**0.0001**	95.6	–	–	0.64	0.2
Wing length	**0.0001**	26.0	0.64	0.2	–	–
10 length	0.49	0.3	0.27	0.6	0.27	0.6
9 length	**0.0001**	13.1	**0.0001**	8.0	**0.0001**	8.0
8 length	**0.0001**	12.7	**0.05**	1.3	**0.05**	1.3

Significant results appear in bold type

**Table 4:** Effects and log utility of models. Juvenile females, adult females, juvenile males and adult males for mass, total length and wing length explained by effects of wing length, total length, mass, length of 8th, 9th, 10th primaries

	Mass		Total length		Wing length	
	Effects	Log Utility	Effects	Log Utility	Effects	Log Utility
Juvenile female						
Mass	–	–	**0.0001**	26.5	**0.0001**	5.2
Total length	**0.0001**	26.5	–	–	**0.028**	1.6
Wing length	**0.0001**	5.2	**0.03**	1.6	–	–
10 length	0.31	0.5	0.6	0.3	0.53	0.3
9 length	0.27	0.6	0.08	1.1	**0.0001**	4.5
8 length	0.43	0.4	0.76	0.1	**0.0001**	26.2
Adult female						
Mass	–	–	**0.0001**	21.7	**0.0001**	6.5
Total length	**0.0001**	21.7	–	–	0.1	1.0
Wing length	**0.0001**	6.5	0.1	1.0	–	–
10 length	**0.005**	2.4	0.87	0.06	0.44	0.4
9 length	0.53	0.3	0.28	0.6	**0.01**	1.9
8 length	**0.002**	2.7	**0.02**	1.8	**0.0001**	10.0
Juvenile male						
Mass	–	–	**0.0001**	21.7	**0.0001**	15.3
Total length	**0.0001**	39.7	–	–	0.74	0.1
Wing length	**0.0001**	15.4	0.74	0.1	–	–
10 length	0.69	20.2	0.2	0.7	**0.006**	2.2
9 length	**0.04**	1.4	**0.04**	1.4	0.56	0.3
8 length	**0.0001**	6.5	**0.02**	1.8	**0.0001**	42.9
Adult male						
Mass	–	–	**0.0001**	52.1	**0.0001**	14.9
Total length	**0.0001**	52.1	–	–	0.43	0.4
Wing length	**0.0001**	14.9	0.43	0.4	–	–
10 length	0.30	0.5	0.49	0.3	0.1	1.0
9 length	0.25	0.6	**0.03**	1.6	**0.03**	1.6
8 length	**0.0001**	3.9	0.42	0.4	0.42	0.4

Significant results appear in bold type

**Fig. 5:**
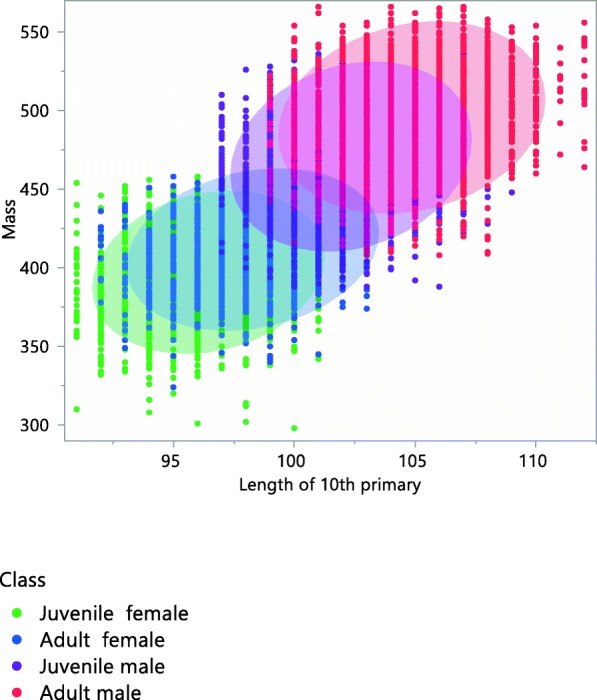
Scaled relationship between wing length (mm) and 10th primary feather length (mm) in Red-legged partridge according to age-sex class (juvenile female, adult female, juvenile male, adult male, ellipses include 90% of observations)

Overall, stronger regression coefficients were found between the length of different primary feathers, followed by coefficients among wing length and primary feather length. Lower coefficients were found in mass and total length (Table 5, Additional files 4 and 5). The relationships among body parts are scaled among age-sex classes, for example, between wing length and the 10th primary feather length (Fig. 5). In the covariance analysis, mass and total length showed a significant interaction that implies slope differences among age and sex classes. We found the same pattern in mass and 9th primary length, and with 8th primary length. However mass and wing length showed no differences in slopes among age and sex classes (Additional files 4, 5, 6 and 7).

**Table 5:** Coefficients of determination between partridge parameters collected from 1998 to 2011

	Mass	Total length	Wing length	10th primary	9th primary	8th primary
Mass	.	**7277**	**8450**	**8368**	**9156**	**7332**
Total length	0.63	.	**6454**	**6317**	**6931**	**5656**
Wing length	0.61	0.53	.	**9769**	**10,622**	**8763**
10th primary	0.56	0.52	0.72	.	**11,120**	**8661**
9th primary	0.54	0.53	0.70	0.85	.	**9782**
8th primary	0.56	0.54	0.79	0.84	0.88	.

The sampled size for each regression appear in bold type

## Discussion

For animal locomotion, the CoM must remain within the limits of stability [43]. In partridges, our results show that the CoM simplified model changes in walking, running and flying and is scaled within age-sex classes. Total length and trunk inclination explains this variation; consequently, total length is important to walking and flying [44]. According to their mass and related to their total length, partridge age-sex classes scale their CoM position with regards to walking and flying [45]. CoM position and body proportions affect mobility; less mass and better balance facilitates escape terrestrially and aerially [46]. Impaired body proportions hamper motion and make individuals more susceptible to predation. Conversely, balanced body structure increases movement and survival [47]. When partridges walk, they bob their head, a back-and-forth movement from the neck, serving as a stabilizing reflex [35]. Head-bobbing allows partridges to scan the ground and sky, searching for food and predators. To obtain food and lower predation probability, partridge flocks require coordination of their individual activity to alternate surveillance and feeding tasks [48] and walking, running and flying as cohesive groups to increase escape efficiency [49]. Accordingly, our findings highlight the potential of body structure to affect survival.

The wing length model is a better descriptor than the total length and mass models for explaining body relations. The wing model is more related to flight, and the total length model is more related to walking-running. The mass model is related to both locomotion types. This suggests that partridge body structure is most conditioned to flying. Relationships with predator–prey body mass ratios influence predation impact [48, 50] since the selection of prey enhances attack success [51, 52] and energetic trade-offs [53]. As a consequence, changes in species proportions [54] and increasing potential to escape predation [55] could condition survival. Wing models in age-sex classes of partridges were better linked to juvenile females than other age-classes, because juvenile females have less mass and a larger surface/mass index than the other age-sex classes [39]. Thus, females have a better conformation than males, and juveniles than adults, for flying but not for walking-running; for walking-running, males and adults have an advantage due to their larger size [47]. Our findings support other studies that found that females quickly develop the ability to move at speeds similar to males to avoid higher rates of predation [5]. Mass limits flight more than running, and hence females and juveniles have proportions that are respectively better adapted to flying than adults and males. The cost of flight increases for larger birds, whereas the cost of terrestrial locomotion increases for smaller birds [12].

Flight feather lengths showed the greatest relationship among one another, followed by the relationship between wing length and feather lengths, and then by the relationship between body mass and total length, with the weakest relationship between body traits and flight feathers [56]. Developing animals are particularly vulnerable to predation. Most predators choose to predate on chicks because of their lower mobility and maneuverability, compared to more experienced and older individuals [57]. In this sense, precocial birds invest great effort in the early development of a body structure that facilitates walking and running. Jump-flight is possible at 6 days old, and short flights occur at 12 days old. The legs and wings work cooperatively for inclined walking, rapid running, take-off and landing [28]. According to our results, there is a moderate relationship among wing length and body mass [58], as well as with primary feather length, which are determinants for flight [26]. Thus, our findings show that wing length is related to the size of other body parts, underscoring the importance of body structure conformation for flight [59].

Our findings show a multiobjective optimization in body proportions as a result of constraints due to conflicts between terrestrial and aerial locomotion. Body part proportions in partridges seem to follow a pattern that could be associated with the behavioral ecology of the species. Certain relationships between the mass and body parts are dissimilar among different age and sex classes, while others are similar (Additional file 4). As a result, all individuals of all ages and sexes do not meet the same terrestrial locomotion requirements, although they meet the same aerodynamic requirements.

## Conclusions

Proportions among body mass, parts, limbs and flight feathers are traits of the partridge body. Wing and flight feathers, mass and CoM relationships are scaled among age-sex classes. According to previous studies, in autumn individuals of all age-sex classes achieve similar motion capacities [60]. The scaled bodies of age-sex classes might provide advantages for group survival [38]. The age-sex scaled size implies a difference in locomotor capacity of individuals, which must be addressed with behavior (e.g., chicks need parental care to survive) [49]. Proportions in body structure may contribute to explaining the success of partridge flocks in obtaining food and reducing predation.

## Additional files

Additional file 1: Changes in the inclination of the trunk and the CoM (Center of Mass) during the transition from walking to running and to flying in partridges. (DOCX 175 kb)

Additional file 2: Mass models. (DOCX 15 kb)

Additional file 3: Total length models. (DOCX 15 kb)

Additional file 4: Covariance analysis for pairs of parameters. (DOCX 26 kb)

Additional file 5: All partridge samples confidence intervals and correlation coefficients. (DOCX 28 kb)

Additional file 6: Multiple regression models of partridge age-sex classes. (DOCX 26 kb)

Additional file 7: Multiple regression models with and without age-sex class. (DOCX 21 kb)

Additional file 8: Multiple regression models with age, sex and reduced feather factors. (DOCX 25 kb)

Additional file 9: Estimation of simplified CoM (Center of Mass) for a section of walking Red-legged partridge according to age-sex classes: adult male, juvenile male, adult female, juvenile female (from left to right). (DOCX 157 kb)
