# Supplementary material for: Body proportions for the facilitation of walking, running and flying: the case of partridges
Source: BMC Evol Biol. 2018 Nov 26;18:176. doi: 10.1186/s12862-018-1295-x (PMC6260763; doi:10.1186/s12862-018-1295-x)
Supplement: Supplementary file 1 — Changes in the inclination of the trunk and the CoM (Center of Mass) during the transition from walking to running and to flying in partridges. (DOCX 175 kb) [file 12862_2018_1295_MOESM1_ESM.docx]

**Additional file 1**. Changes in the inclination of the trunk and the CoM (Center of Mass) during the transition from walking to running and to flying in partridges.
