# Supplementary material for: Body proportions for the facilitation of walking, running and flying: the case of partridges
Source: BMC Evol Biol. 2018 Nov 26;18:176. doi: 10.1186/s12862-018-1295-x (PMC6260763; doi:10.1186/s12862-018-1295-x)
Supplement: Supplementary file 3 — Total length models. (DOCX 15 kb) [file 12862_2018_1295_MOESM3_ESM.docx]

**Additional file 3.** Total length models

**Total length** **models**, juvenile female (JF), adult female (AF), juvenile male (JM), adult male (AM), old (A), young (J), female (F) and male (M) explained by wing length (WL), Mass (MA), the length of the 8^th^ (8), 9^th^ (9) and 10^th^ (10) primaries. Corrected Akaike information criterion (AICc), sample size (N) and p-values.

| **Total length** **models** | AIC_C_ | WL | MA | 10 | 9 | 8 | N |
| --- | --- | --- | --- | --- | --- | --- | --- |
| JF | 5533 | 0.03 | <0.0001 | 0.61 | 0.08 | 0.76 | 811 |
| AF | 4843 | 0.02 | <0.0001 | 0.28 | 0.10 | 0.87 | 717 |
| JM | 6227 | 0.02 | <0.0001 | 0.20 | 0.04 | 0.74 | 896 |
| AM | 10310 | 0.03 | <0.0001 | 0.43 | 0.43 | 0.49 | 1488 |
| A | 15199 | 0.02 | <0.0001 | 0.34 | 0.20 | 0.004 | 2205 |
| J | 11781 | 0.007 | <0.0001 | 0.92 | 0.01 | 0.005 | 1707 |
| F | 10401 | 0.04 | <0.0001 | 0.03 | <0.0001 | 0.38 | 1528 |
| M | 16535 | 0.27 | <0.0001 | 0.05 | <0.0001 | 0.64 | 2384 |
