# Supplementary material for: Body proportions for the facilitation of walking, running and flying: the case of partridges
Source: BMC Evol Biol. 2018 Nov 26;18:176. doi: 10.1186/s12862-018-1295-x (PMC6260763; doi:10.1186/s12862-018-1295-x)
Supplement: Supplementary file 4 — Covariance analysis for pairs of parameters. (DOCX 26 kb) [file 12862_2018_1295_MOESM4_ESM.docx]

**Additional file 4.** Covariance analysis for pairs of parameters, ns = non significant.

|  | All classes  R^2^ | Juvenile female  R^2^ | Adult female  R^2^ | Juvenile male  R^2^ | Adult male  R^2^ |
| --- | --- | --- | --- | --- | --- |
| Mass vs total length | 0.76 | 0.19 | 0.15 | 0.22 | 0.18 |
| Mass vs wing length | 0.73 | 0.09 | 0.07 | 0.09 | 0.08 |
| Mass vs 10 ^th^ primary length | 0.62 | 0.06 | 0.04 | 0.06 | 0.06 |
| Mass vs 9 ^th^ primary length | 0.71 | 0.04 | 0.02 | 0.05 | 0.03 |
| Mass vs 8 ^th^ primary length | 0.71 | 0.04 | 0.02 | 0.04 | 0.04 |
| Total length vs wing length | 0.62 | 0.07 | 0.07 | 0.09 | 0.07 |

|  | Interaction | Juvenile female  b±SD | Adult female  b±SD | Juvenile male  b±SD | Adult male  b±SD |
| --- | --- | --- | --- | --- | --- |
| Mass vs total length | P<0.0004 | 1.21±0.06 | 1.12±0.07 | 1.47±0.06 | 1.39±0.06 |
| Mass vs wing length | ns | 2.47±0.17 | 2.27±0.20 | 2.74±0.18 | 2.62±0.18 |
| Mass vs 10 ^th^ primary length | ns | 0.93±0.07 | 0.60±0.11 | 0.97±0.09 | 0.87±0.08 |
| Mass vs 9 ^th^ primary length | P<0.04 | 2.23±0.2 | 1.57±0.25 | 2.57±0.23 | 2.12±0.22 |
| Mass vs 8 ^th^ primary length | P<0.0001 | 2.06±0.27 | 1.06±0.23 | 2.25±0.30 | 2.19±0.22 |
| Total length vs wing length | P<0.0001 | 0.77±0.07 | 0.72±0.08 | 0.85±0.07 | 0.75±0.06 |

Covariance analysis for pairs of natural logarithm (lg) of parameters, ns = non significant.

|  | All classes  R^2^ | Juvenile female  R^2^ | Adult female  R^2^ | Juvenile male  R^2^ | Adult male  R^2^ |
| --- | --- | --- | --- | --- | --- |
| Lg Mass vs lg total length | 0.76 | 0.17 | 0.15 | 0.22 | 0.18 |
| Lg Mass vs lg wing length | 0.73 | 0.08 | 0.07 | 0.10 | 0.08 |
| Lg Mass vs lg 10 ^th^ primary length | 0.72 | 0.06 | 0.04 | 0.06 | 0.06 |
| Lg Mass vs lg 9 ^th^ primary length | 0.72 | 0.05 | 0.02 | 0.05 | 0.04 |
| Lg Mass vs lg 8 ^th^ primary length | 0.72 | 0.04 | 0.03 | 0.04 | 0.04 |
| Lg total length vs lg wing length | 0.63 | 0.07 | 0.07 | 0.09 | 0.07 |

|  | Interaction | Juvenile female  b±SD | Adult female  b±SD | Juvenile male  b±SD | Adult male  b±SD |
| --- | --- | --- | --- | --- | --- |
| Lg Mass vs lg total length | ns | 1.04±0.05 | 1.13±0.05 | 0.94±0.06 | 1.04±0.05 |
| Lg Mass vs lg wing length | ns | 0.95±0.06 | 0.95±0.06 | 0.86±0.07 | 0.87±0.06 |
| Lg Mass vs lg 10 ^th^ primary length | ns | 0.50±0.05 | 0.50±0.05 | 0.50±0.07 | 0.44±0.05 |
| Lg Mass vs lg 9 ^th^ primary length | ns | 0.58±0.05 | 0.41±0.07 | 0.60±0.06 | 0.49±0.05 |
| Lg Mass vs lg 8 ^th^ primary length | ns | 0.56±0.07 | 0.44±0.06 | 0.55±0.07 | 0.52±0.05 |
| Lg total length vs lg wing length | ns | 0.35±0.03 | 0.32±0.03 | 0.38±0.03 | 0.33±0.03 |
