# Supplementary material for: Body proportions for the facilitation of walking, running and flying: the case of partridges
Source: BMC Evol Biol. 2018 Nov 26;18:176. doi: 10.1186/s12862-018-1295-x (PMC6260763; doi:10.1186/s12862-018-1295-x)
Supplement: Supplementary file 5 — All partridge samples confidence intervals and correlation coefficients. (DOCX 28 kb) [file 12862_2018_1295_MOESM5_ESM.docx]

**Additional file 5.** All partridge samples confidence intervals and correlation coefficients

All partridge samples

|  | **N** |
| --- | --- |
| **Mass** | 9798 |
| **Total length** | 7401 |
| **Wing length** | 11392 |
| **10**^th^ **p. length** | 11710 |
| **9**^th^ **p. length** | 12840 |
| **8**^th^ **p. length** | 10544 |

All partridge correlations

|  | **Mass** | **Total length** | **Wing length** | **10**^th^ **p. length** | **9**^th^ **p. length** | **8**^th^ **p. length** |
| --- | --- | --- | --- | --- | --- | --- |
| **Mass** | 1.0000 | 0.7964 | 0.7841 | 0.7476 | 0.7338 | 0.7481 |
| **Total length** | 0.7964 | 1.0000 | 0.7310 | 0.7193 | 0.7307 | 0.7334 |
| **Wing length** | 0.7841 | 0.7310 | 1.0000 | 0.8468 | 0.8356 | 0.8880 |
| **10**^th^ **p. length** | 0.7476 | 0.7193 | 0.8468 | 1.0000 | 0.9234 | 0.9155 |
| **9**^th^ **p. length** | 0.7338 | 0.7307 | 0.8356 | 0.9234 | 1.0000 | 0.9390 |
| **8**^th^ **p. length** | 0.7481 | 0.7334 | 0.8880 | 0.9155 | 0.9390 | 1.0000 |

All partridge confidence intervals of correlation coefficients

|  |  | **r** | **Lower CI 95%** | **Upper CI 95%** |
| --- | --- | --- | --- | --- |
| **Total length** | **Mass** | 0.7964 | 0.7879 | 0.8047 |
| **Wing length** | **Mass** | 0.7841 | 0.7757 | 0.7922 |
| **Wing length** | **Total length** | 0.7310 | 0.7194 | 0.7422 |
| **10**^th^ **p. length** | **Mass** | 0.7476 | 0.7380 | 0.7569 |
| **10**^th^ **p. length** | **Total length** | 0.7193 | 0.7072 | 0.7310 |
| **10**^th^ **p. length** | **Wing length** | 0.8468 | 0.8411 | 0.8523 |
| **9**^th^ **p. length** | **Mass** | 0.7338 | 0.7243 | 0.7432 |
| **9**^th^ **p. length** | **Total length** | 0.7307 | 0.7196 | 0.7415 |
| **9**^th^ **p. length** | **Wing length** | 0.8356 | 0.8297 | 0.8412 |
| **9**^th^ **p. length** | **10**^th^ **p. length** | 0.9234 | 0.9206 | 0.9261 |
| **8**^th^ **p. length** | **Mass** | 0.7481 | 0.7379 | 0.7580 |
| **8**^th^ **p. length** | **Total length** | 0.7334 | 0.7212 | 0.7453 |
| **8**^th^ **p. length** | **Wing length** | 0.8880 | 0.8835 | 0.8924 |
| **8**^th^ **p. length** | **10**^th^ **p. length** | 0.9155 | 0.9120 | 0.9188 |
| **8**^th^ **p. length** | **9**^th^ **p. length** | 0.9390 | 0.9366 | 0.9413 |

Juvenile female samples

|  | **N** |
| --- | --- |
| **Mass** | 2610 |
| **Total length** | 1802 |
| **Wing length** | 2952 |
| **10**^th^ **p. length** | 3425 |
| **9**^th^ **p. length** | 3417 |
| **8**^th^ **p. length** | 2004 |

Juvenile female correlations

|  | **Mass** | **Total length** | **Wing length** | **10**^th^ **p. length** | **9**^th^ **p. length** | **8**^th^ **p. length** |
| --- | --- | --- | --- | --- | --- | --- |
| **Mass** | 1.0000 | 0.4495 | 0.3119 | 0.1924 | 0.2083 | 0.2178 |
| **Total length** | 0.4495 | 1.0000 | 0.2903 | 0.2522 | 0.2816 | 0.2639 |
| **Wing length** | 0.3119 | 0.2903 | 1.0000 | 0.5098 | 0.5671 | 0.6418 |
| **10**^th^ **p. length** | 0.1924 | 0.2522 | 0.5098 | 1.0000 | 0.8300 | 0.6867 |
| **9**^th^ **p. length** | 0.2083 | 0.2816 | 0.5671 | 0.8300 | 1.0000 | 0.7464 |
| **8**^th^ **p. length** | 0.2178 | 0.2639 | 0.6418 | 0.6867 | 0.7464 | 1.0000 |

Juvenile female confidence intervals of correlation coefficients

|  |  | | **r** | | **Lower CI 95%** | | **Upper CI 95%** | |  |
| --- | --- | --- | --- | --- | --- | --- | --- | --- | --- |
| **Total length** | | **Mass** | | 0.4495 | | 0.4115 | | 0.4858 | |
| **Wing length** | | **Mass** | | 0.3119 | | 0.2741 | | 0.3487 | |
| **Wing length** | | **Total length** | | 0.2903 | | 0.2445 | | 0.3349 | |
| **10**^th^ **p. length** | | **Mass** | | 0.1924 | | 0.1545 | | 0.2297 | |
| **10**^th^ **p. length** | | **Total length** | | 0.2522 | | 0.2077 | | 0.2956 | |
| **10**^th^ **p. length** | | **Wing length** | | 0.5098 | | 0.4822 | | 0.5365 | |
| **9**^th^ **p. length** | | **Mass** | | 0.2083 | | 0.1706 | | 0.2453 | |
| **9**^th^ **p. length** | | **Total length** | | 0.2816 | | 0.2378 | | 0.3243 | |
| **9**^th^ **p. length** | | **Wing length** | | 0.5671 | | 0.5416 | | 0.5915 | |
| **9**^th^ **p. length** | | **10**^th^ **p. length** | | 0.8300 | | 0.8191 | | 0.8403 | |
| **8**^th^ **p. length** | | **Mass** | | 0.2178 | | 0.1676 | | 0.2668 | |
| **8**^th^ **p. length** | | **Total length** | | 0.2639 | | 0.2046 | | 0.3212 | |
| **8**^th^ **p. length** | | **Wing length** | | 0.6418 | | 0.6128 | | 0.6691 | |
| **8**^th^ **p. length** | | **10**^th^ **p. length** | | 0.6867 | | 0.6625 | | 0.7095 | |
| **8**^th^ **p. length** | | **9**^th^ **p. length** | | 0.7464 | | 0.7260 | | 0.7656 | |

Adult female samples

|  | **N** |
| --- | --- |
| **Mass** | 2553 |
| **Total length** | 1867 |
| **Wing length** | 2983 |
| **10**^th^ **p. length** | 3465 |
| **9**^th^ **p. length** | 3429 |
| **8**^th^ **p. length** | 2097 |

Adult female correlations

|  | **Mass** | **Total length** | **Wing length** | **10**^th^ **p. length** | **9**^th^ **p. length** | **8**^th^ **p. length** |
| --- | --- | --- | --- | --- | --- | --- |
| **Mass** | 1.0000 | 0.4025 | 0.2567 | 0.1977 | 0.1558 | 0.1505 |
| **Total length** | 0.4025 | 1.0000 | 0.2663 | 0.2024 | 0.2166 | 0.2528 |
| **Wing length** | 0.2567 | 0.2663 | 1.0000 | 0.5382 | 0.6142 | 0.6542 |
| **10**^th^ **p. length** | 0.1977 | 0.2024 | 0.5382 | 1.0000 | 0.8119 | 0.7636 |
| **9**^th^ **p. length** | 0.1558 | 0.2166 | 0.6142 | 0.8119 | 1.0000 | 0.8889 |
| **8**^th^ **p. length** | 0.1505 | 0.2528 | 0.6542 | 0.7636 | 0.8889 | 1.0000 |

Adult female confidence intervals of correlation coefficients

|  |  | | **r** | **Lower CI 95%** | **Upper CI 95%** |
| --- | --- | --- | --- | --- | --- |
| **Total length** | | **Mass** | 0.4025 | 0.3593 | 0.4440 |
| **Wing length** | | **Mass** | 0.2567 | 0.2112 | 0.3011 |
| **Wing length** | | **Total length** | 0.2663 | 0.2157 | 0.3154 |
| **10**^th^ **p. length** | | **Mass** | 0.1977 | 0.1395 | 0.2546 |
| **10**^th^ **p. length** | | **Total length** | 0.2024 | 0.1386 | 0.2645 |
| **10**^th^ **p. length** | | **Wing length** | 0.5382 | 0.4985 | 0.5757 |
| **9**^th^ **p. length** | | **Mass** | 0.1558 | 0.1078 | 0.2031 |
| **9**^th^ **p. length** | | **Total length** | 0.2166 | 0.1642 | 0.2677 |
| **9**^th^ **p. length** | | **Wing length** | 0.6142 | 0.5851 | 0.6417 |
| **9**^th^ **p. length** | | **10**^th^ **p. length** | 0.8119 | 0.7930 | 0.8291 |
| **8**^th^ **p. length** | | **Mass** | 0.1505 | 0.1056 | 0.1948 |
| **8**^th^ **p. length** | | **Total length** | 0.2528 | 0.2044 | 0.2999 |
| **8**^th^ **p. length** | | **Wing length** | 0.6542 | 0.6293 | 0.6779 |
| **8**^th^ **p. length** | | **10**^th^ **p. length** | 0.7636 | 0.7417 | 0.7839 |
| **8**^th^ **p. length** | | **9**^th^ **p. length** | 0.8889 | 0.8799 | 0.8974 |

Juvenile male samples

|  | **N** |
| --- | --- |
| **Mass** | 2553 |
| **Total length** | 1867 |
| **Wing length** | 2983 |
| **10**^th^ **p. length** | 3465 |
| **9**^th^ **p. length** | 3429 |
| **8**^th^ **p. length** | 2097 |

Juvenile male correlations

|  | **Mass** | **Total length** | **Wing length** | **10**^th^ **p. length** | **9**^th^ **p. length** | **8**^th^ **p. length** |
| --- | --- | --- | --- | --- | --- | --- |
| **Mass** | 1.0000 | 0.4754 | 0.3155 | 0.1928 | 0.2187 | 0.1691 |
| **Total length** | 0.4754 | 1.0000 | 0.2840 | 0.2454 | 0.2974 | 0.2804 |
| **Wing length** | 0.3155 | 0.2840 | 1.0000 | 0.5300 | 0.5859 | 0.6834 |
| **10**^th^ **p. length** | 0.1928 | 0.2454 | 0.5300 | 1.0000 | 0.8447 | 0.6883 |
| **9**^th^ **p. length** | 0.2187 | 0.2974 | 0.5859 | 0.8447 | 1.0000 | 0.7631 |
| **8**^th^ **p. length** | 0.1691 | 0.2804 | 0.6834 | 0.6883 | 0.7631 | 1.0000 |

Juvenile male confidence intervals of correlation coefficients

|  |  | | **r** | **Lower CI 95%** | **Upper CI 95%** |
| --- | --- | --- | --- | --- | --- |
| **Total length** | | **Mass** | 0.4754 | 0.4391 | 0.5102 |
| **Wing length** | | **Mass** | 0.3155 | 0.2773 | 0.3527 |
| **Wing length** | | **Total length** | 0.2840 | 0.2388 | 0.3280 |
| **10**^th^ **p. length** | | **Mass** | 0.1928 | 0.1548 | 0.2303 |
| **10**^th^ **p. length** | | **Total length** | 0.2454 | 0.2019 | 0.2880 |
| **10**^th^ **p. length** | | **Wing length** | 0.5300 | 0.5033 | 0.5557 |
| **9**^th^ **p. length** | | **Mass** | 0.2187 | 0.1809 | 0.2559 |
| **9**^th^ **p. length** | | **Total length** | 0.2974 | 0.2549 | 0.3388 |
| **9**^th^ **p. length** | | **Wing length** | 0.5859 | 0.5614 | 0.6094 |
| **9**^th^ **p. length** | | **10**^th^ **p. length** | 0.8447 | 0.8347 | 0.8541 |
| **8**^th^ **p. length** | | **Mass** | 0.1691 | 0.1184 | 0.2190 |
| **8**^th^ **p. length** | | **Total length** | 0.2804 | 0.2239 | 0.3351 |
| **8**^th^ **p. length** | | **Wing length** | 0.6834 | 0.6579 | 0.7073 |
| **8**^th^ **p. length** | | **10**^th^ **p. length** | 0.6883 | 0.6648 | 0.7105 |
| **8**^th^ **p. length** | | **9**^th^ **p. length** | 0.7631 | 0.7442 | 0.7808 |

Adult male samples

|  | **N** |
| --- | --- |
| **Mass** | 2721 |
| **Total length** | 2210 |
| **Wing length** | 3252 |
| **10**^th^ **p. length** | 3268 |
| **9**^th^ **p. length** | 3719 |
| **8**^th^ **p. length** | 3839 |

Adult male correlations

|  | **Mass** | **Total length** | **Wing length** | **10**^th^ **p. length** | **9**^th^ **p. length** | **8**^th^ **p. length** |
| --- | --- | --- | --- | --- | --- | --- |
| **Mass** | 1.0000 | 0.4394 | 0.2931 | 0.1974 | 0.2076 | 0.1979 |
| **Total length** | 0.4394 | 1.0000 | 0.2782 | 0.2648 | 0.2956 | 0.2864 |
| **Wing length** | 0.2931 | 0.2782 | 1.0000 | 0.5825 | 0.6466 | 0.6975 |
| **10**^th^ **p. length** | 0.1974 | 0.2648 | 0.5825 | 1.0000 | 0.8256 | 0.7820 |
| **9**^th^ **p. length** | 0.2076 | 0.2956 | 0.6466 | 0.8256 | 1.0000 | 0.9012 |
| **8**^th^ **p. length** | 0.1979 | 0.2864 | 0.6975 | 0.7820 | 0.9012 | 1.0000 |

Adult male confidence intervals of correlation coefficients

|  |  | | **r** | **Lower CI 95%** | **Upper CI 95%** |
| --- | --- | --- | --- | --- | --- |
| **Total length** | | **Mass** | 0.4394 | 0.4049 | 0.4728 |
| **Wing length** | | **Mass** | 0.2931 | 0.2556 | 0.3297 |
| **Wing length** | | **Total length** | 0.2782 | 0.2363 | 0.3190 |
| **10**^th^ **p. length** | | **Mass** | 0.1974 | 0.1576 | 0.2366 |
| **10**^th^ **p. length** | | **Total length** | 0.2648 | 0.2218 | 0.3067 |
| **10**^th^ **p. length** | | **Wing length** | 0.5825 | 0.5570 | 0.6068 |
| **9**^th^ **p. length** | | **Mass** | 0.2076 | 0.1701 | 0.2444 |
| **9**^th^ **p. length** | | **Total length** | 0.2956 | 0.2558 | 0.3343 |
| **9**^th^ **p. length** | | **Wing length** | 0.6466 | 0.6254 | 0.6668 |
| **9**^th^ **p. length** | | **10**^th^ **p. length** | 0.8256 | 0.8140 | 0.8365 |
| **8**^th^ **p. length** | | **Mass** | 0.1979 | 0.1609 | 0.2343 |
| **8**^th^ **p. length** | | **Total length** | 0.2864 | 0.2470 | 0.3248 |
| **8**^th^ **p. length** | | **Wing length** | 0.6975 | 0.6791 | 0.7150 |
| **8**^th^ **p. length** | | **10**^th^ **p. length** | 0.7820 | 0.7681 | 0.7952 |
| **8**^th^ **p. length** | | **9**^th^ **p. length** | 0.9012 | 0.8949 | 0.9072 |
