# Supplementary material for: Body proportions for the facilitation of walking, running and flying: the case of partridges
Source: BMC Evol Biol. 2018 Nov 26;18:176. doi: 10.1186/s12862-018-1295-x (PMC6260763; doi:10.1186/s12862-018-1295-x)
Supplement: Supplementary file 6 — Multiple regression models of partridge age-sex classes. (DOCX 26 kb) [file 12862_2018_1295_MOESM6_ESM.docx]

**Additional file 6.** Multiple regression models of partridge age-sex classes

Wing length age-sex classes explained by multiple regression

**Adult female** wing length explained by multiple regression

|  |  |  | F | P |
| --- | --- | --- | --- | --- |
| R^2^ | 0.44 | ANOVA | 112.06 | <.0001 |
| N | 717 | Lak of fit | 1.25 | 0.55 |
| AICc | 3048.09 |  |  |  |

|  | log utility | P | VIF |
| --- | --- | --- | --- |
| 8 length | 9.92 | 0.00000 | 5.07 |
| Mass | 6.45 | 0.00000 | 1.21 |
| 9 length | 1.87 | 0.01347 | 6.27 |
| Total length | 0.99 | 0.10344 | 1.21 |
| 10 length | 0.36 | 0.44109 | 3.03 |

**Adult male** wing length explained by multiple regression

|  |  |  | F | P |
| --- | --- | --- | --- | --- |
| R^2^ | 0.22 | ANOVA | 82.05 | <.0001 |
| N | 1488 | Lak of fit | 0.81 | 0.78 |
| AICc | 10309.96 |  |  |  |

|  | log utility | P | VIF |
| --- | --- | --- | --- |
| 8 length | 41.17 | 0.00000 | 5.05 |
| Mass | 14.79 | 0.00000 | 1.22 |
| 10 length | 1.02 | 0.09606 | 2.92 |
| Total length | 0.36 | 0.43330 | 1.23 |
| 9 length | 0.09 | 0.81077 | 6.22 |

**Juvenile female** wing length explained by multiple regression

|  |  |  | F | P |
| --- | --- | --- | --- | --- |
| R^2^ | 0.43 | ANOVA | 124.29 | <.0001 |
| N | 811 | Lak of fit | 2.57 | 0.99 |
| AICc | 3488.19 |  |  |  |

|  | log utility | P | VIF |
| --- | --- | --- | --- |
| 8 length | 25.98 | 0.00000 | 2.21 |
| Mass | 5.11 | 0.00001 | 1.23 |
| 9 length | 4.48 | 0.00003 | 4.06 |
| Total length | 1.54 | 0.02900 | 1.27 |
| 10 length | 0.28 | 0.52954 | 3.23 |

**Juvenile male** wing length explained by multiple regression

|  |  |  | F | P |
| --- | --- | --- | --- | --- |
| R^2^ | 0.52 | ANOVA | 190.57 | <.0001 |
| N | 896 | Lak of fit | 3.71 | 0.24 |
| AICc | 3922.99 |  |  |  |

|  | log utility | P | VIF |
| --- | --- | --- | --- |
| 8 length | 42.61 | 0.00000 | 2.42 |
| Mass | 15.25 | 0.00000 | 1.33 |
| 10 length | 2.21 | 0.00610 | 3.38 |
| 9 length | 0.25 | 0.56265 | 4.46 |
| Total length | 0.13 | 0.73929 | 1.36 |

Mass age-sex classes explained by multiple regression

**Adult female** mass explained by multiple regression

|  |  |  | F | P |
| --- | --- | --- | --- | --- |
| R^2^ | 0.21 | ANOVA | 36.56 | <.0001 |
| N | 717 | Lak of fit | 1.48 | 0.13 |
| AICc | 6394.20 |  |  |  |

|  | log utility | P | VIF |
| --- | --- | --- | --- |
| Total length | 21.49 | 0.00000 | 1.06 |
| Wing length | 6.45 | 0.00000 | 1.72 |
| 8 length | 2.71 | 0.00194 | 5.31 |
| 10 length | 2.33 | 0.00464 | 3.00 |
| 9 length | 0.28 | 0.52839 | 6.33 |

**Adult male** mass explained by multiple regression

|  |  |  | F | P |
| --- | --- | --- | --- | --- |
| R^2^ | 0.22 | ANOVA | 81.35 | <.0001 |
| N | 1488 | Lak of fit | 1.30 | 0.10 |
| AICc | 13780.27 |  |  |  |

|  | log utility | P | VIF |
| --- | --- | --- | --- |
| Total length | 51.90 | 0.00000 | 1.09 |
| Wing length | 14.79 | 0.00000 | 1.84 |
| 8 length | 3.89 | 0.00013 | 2.92 |
| 9 length | 0.61 | 0.24649 | 6.22 |
| 10 length | 0.52 | 0.30547 | 5.66 |

**Juvenile female** mass explained by multiple regression

|  |  |  | F | P |
| --- | --- | --- | --- | --- |
| R^2^ | 0.20 | ANOVA | 41.21 | <.0001 |
| N | 811 | Lak of fit | 0.81 | 0.78 |
| AICc | 7231.44 |  |  |  |

|  | log utility | P | VIF |
| --- | --- | --- | --- |
| Total length | 26.30 | 0.00000 | 1.11 |
| Wing length | 5.11 | 0.00001 | 1.73 |
| 9 length | 0.56 | 0.27320 | 4.14 |
| 10 length | 0.51 | 0.30984 | 3.22 |
| 8 length | 0.37 | 0.42851 | 2.55 |

**Juvenile male** mass explained by multiple regression

|  |  |  | F | P |
| --- | --- | --- | --- | --- |
| R^2^ | 0.30 | ANOVA | 76.64 | <.0001 |
| N | 896 | Lak of fit | 0.74 | 0.87 |
| AICc | 8224.79 |  |  |  |

|  | log utility | P | VIF |
| --- | --- | --- | --- |
| Total length | 39.39 | 0.00000 | 1.11 |
| Wing length | 15.25 | 0.00001 | 1.92 |
| 8 length | 6.49 | 0.27320 | 2.91 |
| 9 length | 1.37 | 0.30984 | 4.44 |
| 10 length | 0.16 | 0.42851 | 3.41 |

Total length age-sex classes explained by multiple regression

**Adult female** total length explained by multiple regression

|  |  |  | F | P |
| --- | --- | --- | --- | --- |
| R^2^ | 0.18 | ANOVA | 30.09 | <.0001 |
| N | 717 | Lak of fit | 0.66 | 0.79 |
| AICc | 4843.29 |  |  |  |

|  | log utility | P | VIF |
| --- | --- | --- | --- |
| Mass | 21.49 | 0.00000 | 1.10 |
| 8 length | 1.76 | 0.01757 | 5.35 |
| Wing length | 0.99 | 0.10344 | 1.78 |
| 9 length | 0.55 | 0.27939 | 6.32 |
| 10 length | 0.06 | 0.86791 | 3.03 |

**Adult male** total length explained by multiple regression

|  |  |  | F | P |
| --- | --- | --- | --- | --- |
| R^2^ | 0.22 | ANOVA | 82.05 | <.0001 |
| N | 1488 | Lak of fit | 0.81 | 0.78 |
| AICc | 10309.96 |  |  |  |

|  | log utility | P | VIF |
| --- | --- | --- | --- |
| Mass | 51.90 | 0.00000 | 1.09 |
| 9 length | 1.55 | 0.02823 | 6.21 |
| 8 length | 0.37 | 0.42530 | 5.71 |
| Wing length | 0.36 | 0.43330 | 1.92 |
| 10 length | 0.31 | 0.48983 | 2.92 |

**Juvenile female** total length explained by multiple regression

|  |  |  | F | P |
| --- | --- | --- | --- | --- |
| R^2^ | 0.22 | ANOVA | 44.56 | <.0001 |
| N | 811 | Lak of fit | 5.55 | 0.05 |
| AICc | 5532.66 |  |  |  |

|  | log utility | P | VIF |
| --- | --- | --- | --- |
| Mass | 26.30 | 0.00000 | 1.09 |
| Wing length | 1.54 | 0.02900 | 1.76 |
| 9 length | 1.11 | 0.07819 | 4.13 |
| 10 length | 0.22 | 0.60916 | 3.23 |
| 8 length | 0.12 | 0.76321 | 2.55 |

**Juvenile male** total length explained by multiple regression

|  |  |  | F | P |
| --- | --- | --- | --- | --- |
| R^2^ | 0.26 | ANOVA | 63.23 | <.0001 |
| N | 896 | Lak of fit | 1.50 | 0.39 |
| AICc | 6226.65 |  |  |  |

|  | log utility | P | VIF |
| --- | --- | --- | --- |
| Mass | 39.39 | 0.00000 | 1.17 |
| 8 length | 1.76 | 0.01730 | 2.98 |
| 9 length | 1.42 | 0.03806 | 4.44 |
| 10 length | 0.69 | 0.20255 | 3.40 |
| Wing length | 0.13 | 0.73929 | 2.07 |
