# Supplementary material for: Body proportions for the facilitation of walking, running and flying: the case of partridges
Source: BMC Evol Biol. 2018 Nov 26;18:176. doi: 10.1186/s12862-018-1295-x (PMC6260763; doi:10.1186/s12862-018-1295-x)
Supplement: Supplementary file 7 — Multiple regression models with and without age-sex class. (DOCX 21 kb) [file 12862_2018_1295_MOESM7_ESM.docx]

**Additional file 7.** Multiple regression models with and without age-sex class

With age-sex class

Wing length explained by multiple regression (with age-sex class)

|  |  |  | F | P |
| --- | --- | --- | --- | --- |
| R^2^ | 0.84 | ANOVA | 2542.84 | <.0001 |
| N | 3912 | Lak of fit | 2.09 | 0.08 |
| AICc | 17048.63 |  |  |  |

|  | log utility | P | VIF |
| --- | --- | --- | --- |
| 8 length | 116.43 | 0.00000 | 11.81 |
| Class | 49.91 | 0.00000 | 2.88/4.68/6.29 |
| Mass | 38.98 | 0.00000 | 4.27 |
| 9 length | 3.65 | 0.00022 | 20.21 |
| 10 length | 1.94 | 0.01153 | 9.44 |
| Total length | 1.61 | 0.02477 | 3.25 |

Mass explained by multiple regression (with age-sex class)

|  |  |  | F | P |
| --- | --- | --- | --- | --- |
| R^2^ | 0.77 | ANOVA | 1692.46 | <.0001 |
| N | 3912 | Lak of fit | 1.07 | 0.33 |
| AICc | 35673.00 |  |  |  |

|  | log utility | P | VIF |
| --- | --- | --- | --- |
| Class | 166.97 | 0.00000 | 2.63/4.30/5.52 |
| Total length | 137.15 | 0.00000 | 2.78 |
| Wing length | 38.98 | 0.00000 | 5.94 |
| 8 length | 10.30 | 0.00000 | 13.37 |
| 9 length | 1.27 | 0.05428 | 20.26 |
| 10 length | 1.09 | 0.08103 | 9.47 |

Total length explained by multiple regression (with age-sex class)

|  |  |  | F | P |
| --- | --- | --- | --- | --- |
| R^2^ | 0.69 | ANOVA | 1101.74 | <.0001 |
| N | 3912 | Lak of fit | 0.94 | 0.63 |
| AICc | 26906.11 |  |  |  |

|  | log utility | P | VIF |
| --- | --- | --- | --- |
| Mass | 137.15 | 0.00000 | 3.81 |
| Class | 18.11 | 0.00000 | 2.98/4.59/6.19 |
| 9 length | 2.64 | 0.00229 | 20.23 |
| 8 length | 2.11 | 0.00773 | 13.50 |
| Wing length | 1.61 | 0.02477 | 6.20 |
| 10 length | 0.06 | 0.88078 | 9.48 |

Without age-sex class

Wing length and sex explained by multiple regression (without age-sex class)

|  |  |  | F | P |
| --- | --- | --- | --- | --- |
| R^2^ | 0.84 | ANOVA | 3286.64 | <.0001 |
| N | 3912 | Lak of fit | 2.15 | 0.08 |
| AICc | 17148.11 |  |  |  |

|  | log utility | P | VIF |
| --- | --- | --- | --- |
| 8 length | 139.96 | 0.00000 | 11.25 |
| Mass | 80.88 | 0.00000 | 3.34 |
| Age | 29.74 | 0.00000 | 2.29 |
| Total length | 3.73 | 0.00018 | 3.18 |
| 10 length | 3.55 | 0.00028 | 9.33 |
| 9 length | 3.15 | 0.00071 | 20.17 |

Mass and sex explained by multiple regression (without age-sex class)

|  |  |  | F | P |
| --- | --- | --- | --- | --- |
| R^2^ | 0.73 | ANOVA | 1737.94 | <.0001 |
| N | 3912 | Lak of fit | 1.13 | 0.99 |
| AICc | 26906.11 |  |  |  |

|  | log utility | P | VIF |
| --- | --- | --- | --- |
| Total length | 231.94 | 0.00000 | 2.44 |
| Wing length | 80.88 | 0.00000 | 5.51 |
| 10 length | 6.72 | 0.00000 | 9.30 |
| 8 length | 2.98 | 0.00104 | 13.20 |
| Age | 1.98 | 0.01037 | 2.34 |
| 9 length | 1.09 | 0.08128 | 20.21 |

Total length and sex explained by multiple regression (without age-sex class)

|  |  |  | F | P |
| --- | --- | --- | --- | --- |
| R^2^ | 0.69 | ANOVA | 1428.37 | <.0001 |
| N | 717 | Lak of fit | 0.99 | 0.56 |
| AICc | 26906.11 |  |  |  |

|  | log utility | P | VIF |
| --- | --- | --- | --- |
| Mass | 231.94 | 0.00000 | 2.80 |
| 8 length | 4.20 | 0.00006 | 13.19 |
| Wing length | 3.73 | 0.00018 | 6.30 |
| Age | 2.93 | 0.00118 | 2.36 |
| 9 length | 2.29 | 0.00514 | 20.19 |
| 10 length | 0.36 | 0.43345 | 9.36 |
