# Supplementary material for: Body proportions for the facilitation of walking, running and flying: the case of partridges
Source: BMC Evol Biol. 2018 Nov 26;18:176. doi: 10.1186/s12862-018-1295-x (PMC6260763; doi:10.1186/s12862-018-1295-x)
Supplement: Supplementary file 8 — Multiple regression models with age, sex and reduced feather factors. (DOCX 25 kb) [file 12862_2018_1295_MOESM8_ESM.docx]

**Additional file 8**. Multiple regression models with age, sex and reduced feather factors

Wing length and age explained by multiple regression

|  |  |  | F | P |
| --- | --- | --- | --- | --- |
| R^2^ | 0.84 | ANOVA | 3314.07 | <.0001 |
| N | 3912 | Lak of fit | 0.53 | 0.98 |
| AICc | 17148.11 |  |  |  |

|  | log utility | P | VIF |
| --- | --- | --- | --- |
| 8 length | 134.21 | 0.00000 | 11.37 |
| sex | 35.67 | 0.00000 | 4.30 |
| Mass | 34.97 | 0.00000 | 4.23 |
| 10 length | 8.02 | 0.00000 | 8.27 |
| 9 length | 1.46 | 0.03503 | 12.10 |
| Total length | 1.01 | 0.09959 | 3.23 |

Mass and age explained by multiple regression

|  |  |  | F | P |
| --- | --- | --- | --- | --- |
| R^2^ | 0.72 | ANOVA | 2211.67 | <.0001 |
| N | 3912 | Lak of fit | 1.13 | 0.16 |
| AICc | 35731.47 |  |  |  |

|  | log utility | P | VIF |
| --- | --- | --- | --- |
| sex | 156.34 | 0.00000 | 3.73 |
| Total length | 143.71 | 0.00000 | 2.73 |
| Wing length | 34.97 | 0.00000 | 5.85 |
| 8 length | 12.84 | 0.00000 | 13.11 |
| 9 length | 10.94 | 0.00000 | 11.97 |
| 10 length | 0.08 | 0.83594 | 8.34 |

Total length and sex explained by multiple regression

|  |  |  | F | P |
| --- | --- | --- | --- | --- |
| R^2^ | 0.69 | ANOVA | 1451.99 | <.0001 |
| N | 717 | Lak of fit | 0.97 | 0.58 |
| AICc | 26934.96 |  |  |  |

|  | log utility | P | VIF |
| --- | --- | --- | --- |
| Mass | 143.71 | 0.00000 | 3.72 |
| 9 length | 16.14 | 0.00000 | 11.90 |
| sex | 12.84 | 0.00000 | 4.42 |
| 10 length | 1.55 | 0.02824 | 8.33 |
| 8 length | 1.35 | 0.04427 | 13.28 |
| Wing length | 1.00 | 0.09959 | 6.09 |

Wing length explained by multiple regression 8^th^ primary feather

|  |  |  | F | P |
| --- | --- | --- | --- | --- |
| R^2^ | 0.83 | ANOVA | 7748.77 | <.0001 |
| N | 4913 | Lak of fit | 0.99 | 0.52 |
| AICc | 21860.53 |  |  |  |

|  | log utility | P | VIF |
| --- | --- | --- | --- |
| 8 length | 740.01 | 0.00000 | 3.18 |
| Mass | 121.86 | 0.00000 | 3.09 |
| Total length | 3.18 | 0.00067 | 2.59 |

Wing length explained by multiple regression 9^th^ primary feather

|  |  |  | F | P |
| --- | --- | --- | --- | --- |
| R^2^ | 0.77 | ANOVA | 6587.77 | <.0001 |
| N | 5497 | Lak of fit | 0.95 | 0.83 |
| AICc | 37852.32 |  |  |  |

|  | log utility | P | VIF |
| --- | --- | --- | --- |
| 9 length | 549.27 | 0.00000 | 2.48 |
| Mass | 174.58 | 0.00000 | 3.11 |
| Total length | 10.71 | 0.00000 | 3.14 |

Wing length explained by multiple regression 10^th^ primary feather

|  |  |  | F | P |
| --- | --- | --- | --- | --- |
| R^2^ | 0.77 | ANOVA | 6213.50 | <.0001 |
| N | 5464 | Lak of fit | 1.09 | 0.06 |
| AICc | 25805.33 |  |  |  |

|  | log utility | P | VIF |
| --- | --- | --- | --- |
| 10 length | 526.29 | 0.00000 | 2.48 |
| Mass | 121.84 | 0.00000 | 3.30 |
| Total length | 20.31 | 0.00000 | 3.08 |

Mass explained by multiple regression 8^th^ primary feather

|  |  |  | F | P |
| --- | --- | --- | --- | --- |
| R^2^ | 0.68 | ANOVA | 3867.66 | <.0001 |
| N | 5464 | Lak of fit | 1.09 | 0.04 |
| AICc | 37852.32 |  |  |  |

|  | log utility | P | VIF |
| --- | --- | --- | --- |
| Total length | 298.68 | 0.00000 | 2.34 |
| Wing length | 121.86 | 0.00000 | 5.12 |
| 8 length | 1.56 | 0.02738 | 5.17 |

Mass explained by multiple regression 9^th^ primary feather

|  |  |  | F | P |
| --- | --- | --- | --- | --- |
| R^2^ | 0.68 | ANOVA | 4281.06 | <.0001 |
| N | 5497 | Lak of fit | 1.05 | 0.15 |
| AICc | 37852.32 |  |  |  |

|  | log utility | P | VIF |
| --- | --- | --- | --- |
| Total length | 353.78 | 0.00000 | 2.41 |
| Wing length | 174.58 | 0.00000 | 3.78 |
| 9 length | 5.96 | 0.00000 | 3.78 |

Mass explained by multiple regression 10^th^ primary feather

|  |  |  | F | P |
| --- | --- | --- | --- | --- |
| R^2^ | 0.73 | ANOVA | 4822.61 | <.0001 |
| N | 5464 | Lak of fit | 1.18 | <.0001 |
| AICc | 50884.09 |  |  |  |

|  | log utility | P | VIF |
| --- | --- | --- | --- |
| Total length | 336.37 | 0.00000 | 2.35 |
| Wing length | 121.84 | 0.00000 | 3.98 |
| 10 length | 18.14 | 0.00000 | 3.81 |

Total length explained by multiple regression 8^th^ primary feather

|  |  |  | F | P |
| --- | --- | --- | --- | --- |
| R^2^ | 0.68 | ANOVA | 3867.66 | <.0001 |
| N | 5464 | Lak of fit | 1.09 | 0.04 |
| AICc | 37852.32 |  |  |  |

|  | log utility | P | VIF |
| --- | --- | --- | --- |
| Mass | 298.68 | 0.00000 | 2.69 |
| 8 length | 53.84 | 0.00000 | 4.92 |
| Wing length | 3.18 | 0.00067 | 5.72 |

Total length explained by multiple regression 9^th^ primary feather

|  |  |  | F | P |
| --- | --- | --- | --- | --- |
| R^2^ | 0.68 | ANOVA | 4281.06 | <.0001 |
| N | 5497 | Lak of fit | 1.05 | 0.15 |
| AICc | 37852.32 |  |  |  |

|  | log utility | P | VIF |
| --- | --- | --- | --- |
| Mass | 353.78 | 0.00000 | 2.71 |
| 9 length | 81.54 | 0.00000 | 3.56 |
| Wing length | 10.71 | 0.00000 | 4.29 |

Total length explained by multiple regression 10^th^ primary feather

|  |  |  | F | P |
| --- | --- | --- | --- | --- |
| R^2^ | 0.68 | ANOVA | 3867.66 | <.0001 |
| N | 5464 | Lak of fit | 1.09 | 0.04 |
| AICc | 37852.32 |  |  |  |

|  | log utility | P | VIF |
| --- | --- | --- | --- |
| Mass | 336.37 | 0.00000 | 2.75 |
| 10 length | 38.32 | 0.00000 | 3.75 |
| Wing length | 20.31 | 0.00000 | 4.34 |
