# Supplementary material for: Body proportions for the facilitation of walking, running and flying: the case of partridges
Source: BMC Evol Biol. 2018 Nov 26;18:176. doi: 10.1186/s12862-018-1295-x (PMC6260763; doi:10.1186/s12862-018-1295-x)
Supplement: Supplementary file 9 — Estimation of simplified CoM (Center of Mass) for a section of walking Red-legged partridge according to age-sex classes: adult male, juvenile male, adult female, juvenile female (from left to right). (DOCX 157 kb) [file 12862_2018_1295_MOESM9_ESM.docx]

**Additional file 9.** Estimation of simplified CoM (Center of Mass) for a section of walking Red-legged partridge according to age-sex classes: adult male, juvenile male, adult female, juvenile female (from left to right).
